# Supplementary material for: Associations between the Global Diet Quality Score and risk of type 2 diabetes: Tehran lipid and glucose study
Source: PLoS One. 2025 Jan 15;20(1):e0313886. doi: 10.1371/journal.pone.0313886 (PMC11734924; doi:10.1371/journal.pone.0313886)
Supplement: S1 Table — (DOCX) [file pone.0313886.s001.docx]

**Supplementary Table 1.** **Food group components, classification, and point values of the Global Diet Quality Score (GDQS)**

| **Healthy food groups** | | | Classification of consumed amount (gr/day) | | | | Score | | | |
| --- | --- | --- | --- | --- | --- | --- | --- | --- | --- | --- |
| Citrus fruits | | Grapefruit, Orange, Tangerine, Sweet lemon, Lemon | <24 | 24-69 | >69 |  | 0 | 1 | 2 |  |
| Deep orange fruits | | Cantaloupe, Apricot, Persimmon, Dried fruits | <25 | 25-123 | >123 |  | 0 | 1 | 2 |  |
| Other fruits | | Melon, watermelon, Pear, cherries, apple, peach, Prunus, Nectarines, Fresh figs, Dried figs, Grape, Kiwi, Pomegranate, Date, Plums (yellow and red), Strawberry, Banana, Raisins, Fresh berries, Dried berries | <27 | 27-107 | >107 |  | 0 | 1 | 2 |  |
| Dark green leafy vegetables | | Shredded lettuce, Fresh herbs, Cooked vegetables (soup, rice, etc.), Boiled Celery, Cooked spinach | <13 | 13-37 | >37 |  | 0 | 2 | 4 |  |
| Cruciferous vegetables | | Cabbage varieties, Turnip | <13 | 13-36 | >36 |  | 0 | 0.25 | 0.5 |  |
| Deep orange vegetables | | Pumpkin | <9 | 9-45 | >45 |  | 0 | 0.25 | 0.5 |  |
| Other vegetables | | Tomato, cucumber, Stewed pumpkin, Baked Eggplant, Green peas cooked, Green beans cooked, Bell peppers, Small green pepper, Baked mushrooms | <23 | 23-114 | >114 |  | 0 | 0.25 | 0.5 |  |
| Legumes | | Lentil, Beans, Peas, Baked beans, Soybean, Mung, Cotyledon | <9 | 9-42 | >42 |  | 0 | 2 | 4 |  |
| Deep orange tubers | | Raw carrots, Cooked carrots, | <12 | 12-63 | >63 |  | 0 | 0.25 | 0.5 |  |
| Nuts and seeds | | Peanut, Almond, Walnut, Pistachios, Hazelnut, Seeds (watermelon, pumpkin, sunflower) | <7 | 7-13 | >13 |  | 0 | 2 | 4 |  |
| Whole grains | | Sangak bread, barbari bread, corn | <8 | 8-13 | >13 |  | 0 | 1 | 2 |  |
| Liquid oils | | Oil, Olive oil, Mayonnaise, olive | <2 | 2-7.5 | >7.5 |  | 0 | 1 | 2 |  |
| Fish and shellfish | | Fish (except tuna) stating the type, Tuna (canned) | <14 | 14-71 | >71 |  | 0 | 1 | 2 |  |
| Poultry and game meat | | Hen and Chickens | <16 | 16-44 | >44 |  | 0 | 1 | 2 |  |
| Low-fat dairy | | Low-fat milk (less than 2%), Regular yogurt | <33 | 33-132 | >132 |  | 0 | 1 | 2 |  |
| Eggs | | egg | <6 | 6-32 | >32 |  | 0 | 1 | 2 |  |
| **Unhealthy in excessive amounts of food groups** | | |  |  |  |  |  |  |  |  |
| High-fat dairy | | Whole milk (greater or equal to 2%), Ooze Yogurt, Cheese, Chocolate milk, Dough, Dried whey | <35 | 35-142 | >142-734 | >734 | 0 | 1 | 2 | 0 |
| Red meat | | Beef or calf, lamb meat, Ground beef, Heart, liver and kidney, Tripe and Rennet, Tongue, Brain, Head, Leg | <9 | 9-46 | >46 |  | 0 | 1 | 0 |  |
| **Unhealthy food groups** | | |  |  |  |  |  |  |  |  |
| Processed meat | Hamburger, Kielbasa, Sausage, Pizza | | <9 | 9-30 | >30 |  | 2 | 1 | 0 |  |
| Refined grains and baked goods | Lavash bread, Barbari bread, Taftoon bread, Baguette bread, Cooked rice, Baked vermicelli, Ash noodles, Crackers (crispy biscuits), Cooked barley or bulgur | | <7 | 7-33 | >33 |  | 2 | 1 | 0 |  |
| Sweets and ice cream | Cookies (stating the type), Types of cakes, Canned fruits, Sugar cubes, comfit, Sugar, Honey, jams, Dried sweets, creams sweet, Gaz, Sohan, Chocolate, Candy, Homemade Halva, Traditional ice cream, Non-traditional ice cream | | <13 | 13-37 | >37 |  | 2 | 1 | 0 |  |
| Sugar-sweetened beverages | Industrial or cola beverages | | <57 | 57-180 | >180 |  | 2 | 1 | 0 |  |
| Juice | Packaged fruit juices, Lemon juice, Orange juice, Apple juice, Cantaloupe juice | | <36 | 36-144 | >144 |  | 2 | 1 | 0 |  |
| White roots and tubers | Potato flour or cassava flour | | <27 | 27-107 | >107 |  | 2 | 1 | 0 |  |
| Purchased deep-fried foods | Deep fried foods | | <9 | 9-45 | >45 |  | 2 | 1 | 0 |  |
