# Supplementary material for: Associations between the Global Diet Quality Score and risk of type 2 diabetes: Tehran lipid and glucose study
Source: PLoS One. 2025 Jan 15;20(1):e0313886. doi: 10.1371/journal.pone.0313886 (PMC11734924; doi:10.1371/journal.pone.0313886)
Supplement: S2 Table — (DOCX) [file pone.0313886.s002.docx]

**Supplementary Table 2. The number (percentage) of participants according to quartiles of food group scores and based on quartiles of global diet quality score (GDQS)**

| variable | Q1  n(%) | Q2  n(%) | Q3  n(%) | Q4  n(%) | P value |
| --- | --- | --- | --- | --- | --- |
| Citrus fruits | 382 (26) | 796 (54) | 1068 (67) | 1208 (85.6) | <0.001 |
| Deep orange fruits | 10 (0.7) | 41(2.8) | 32 (2) | 76 (5.4 ) | <0.001 |
| Other fruits | 747 (50.9) | 1215(82.5) | 1463 (91.7) | 1386 (98.2) | <0.001 |
| Dark green leafy vegetables | 378 (25.7) | 884 (60) | 1305 (81.8) | 1333 (94.5) | <0.001 |
| Cruciferous vegetables | 18 (1.2) | 38 (2.6) | 60 (3.8) | 123 (8.7) | <0.001 |
| Deep orange vegetables | 0 (0) | 1 (0.1) | 1 (0.1) | 3 (0.2) | <0.001 |
| Other vegetables | 722 (49.1) | 1173 (79.6) | 1356 (85) | 1329 (94.2) | <0.001 |
| Legumes | 183 (12.5) | 420 (28.5) | 672 (42.1) | 951 (67.4) | <0.001 |
| Deep orange tubers | 13 (0.9) | 35 (2.4) | 47 (2.9) | 150 (10.6) | <0.001 |
| Nuts and seeds | 48 (3.3) | 78 (5.3) | 283 (17.7) | 663 (47) | <0.001 |
| Whole grains | 1280 (87.1) | 1390 (94.4) | 1547 (97) | 1392 (98.7) | <0.001 |
| Liquid oils | 604 (41.1) | 830 (56.3) | 1015 (63.6) | 1098 (77.8) | <0.001 |
| Fish and shellfish | 4 (0.3) | 2 (0.1) | 5 (0.3) | 11 (0.8) | <0.001 |
| Poultry and game meat | 131 (8.9) | 193 (13.1) | 273 (17.1) | 399 (28.3) | <0.001 |
| Low-fat dairy | 517 (35.2) | 812 (55.1) | 1103 (69.2) | 1126 (79.8) | <0.001 |
| Eggs | 75 (5.2) | 100 (6.8) | 135 (8.5) | 182 (12.9) | <0.001 |
| High-fat dairy | 964 (65.6) | 1198 (81.3) | 1375 (86.2) | 1303 (96.2) | <0.001 |
| Red meat | 853 (58.1) | 1051 (71.4) | 1218 (76.4) | 1165 (82.6) | <0.001 |
| Processed meat | 564 (38.4) | 562 (38.2) | 710 (44.5) | 686 (48.6) | <0.001 |
| Refined grains and baked goods | 2 (0.1) | 2 (0.1) | 2 (0.1) | 1 (0.1) | 0.95 |
| Sweets and ice cream | 59 (4) | 39 (2.6) | 37 (2.3) | 52 (3.7) | 0.02 |
| Sugar-sweetened beverages juice | 1079 (73.5) | 1174 (79.7) | 1365 (85.6) | 1284 (91) | <0.001 |
| Juice | 1094 (74.5) | 976 (66.3) | 1098 (68.8) | 897 (63.6) | <0.001 |
| White roots and tubers | 687 (46.8) | 527 (35.8) | 502 (31.5) | 363 (25.7) | <0.001 |
| Purchase deep-fried foods | 834 (56.8) | 872 (59.2) | 1045 (65.6) | 965 (68.4) | <0.001 |
