# Supplementary material for: Associations between the Global Diet Quality Score and risk of type 2 diabetes: Tehran lipid and glucose study
Source: PLoS One. 2025 Jan 15;20(1):e0313886. doi: 10.1371/journal.pone.0313886 (PMC11734924; doi:10.1371/journal.pone.0313886)
Supplement: Attachment — Submitted filename: Response to reviwers comments.docx [file pone.0313886.s003.docx]

Dear Dr. Meisam Akhlaghdoust

I would like to express my gratitude for your valuable time and thoughtful comments on our manuscript “Associations between the Global Diet Quality Score and risk of Type 2 Diabetes: Tehran Lipid and Glucose Study”. Your insights have been instrumental in refining our work.

In response to your suggestions, we have diligently revised the manuscript. To facilitate your review, we have provided a point-by-point summary of the requested changes, and the modifications are highlighted in the submitted file. We are hopeful that the revised manuscript now aligns with the standards for publication in “PLOS ONE” Your interest in our research and the opportunity to enhance its quality are greatly appreciated. We remain open to any additional feedback or further revisions that you may recommend ensuring the manuscript meets the journal’s criteria .Once again, thank you for your commitment to advancing scientific discourse, and we look forward to your feedback on the revised version.

Please find the below comments/questions by the editor/reviewers (in black color) and our response/clarification/explanation to the comments and questions (in blue color).

Best regards,

**Parvin Mirmiran, Prof.**

E-mail: parvin.mirmiran@sbmu.ac.ir

Parvin.mirmiran@gmail.com

**Reviewer #1:**

**I would like to thank the authors to design this valuable study. However, there are some comments as follows:**

**Please revise the manuscript in terms of English language and grammer.**

**Abstract:
Background: Please use association instead of relation. Please revise it in the whole manuscript.
Reply: Agreed and corrected in all of the manuscript.**

**Methods: In this secondary analysis, we included elective adult participants (n=5948) from the Tehran Lipid and Glucose Study. Please mention the Phase of the TLGS you used the data.
Reply: Agreed and corrected**

**In this secondary analysis, we included elective adult participants (n=5948) from the third and fourth phase of the Tehran Lipid and Glucose Study. Participants checked out until the sixth phase with an average follow-up of 6.65 years.**

**Please mention the components of GDQS score.**

**Reply: The GDQS were calculated including healthy and unhealthy food group scores.**

**Food groups are divided into 16 healthy including kinds of fruits and vegetables, legumes, nuts and seeds, whole grain, fish, poultry, liquid oil, low-fat dairy, and egg; 7 unhealthy including processed meats, refined grains, sweets, sugar-sweetened beverages, potato or cassava flour, juice, and deep fried foods; and two unhealthy when consumed more than recommendation including red meat and high-fat dairy. (page 4, line 61-64)**

**Results:
Please report mean(SD) instead of mean±SD**

**Reply: Agreed and corrected**

**Please report the 6 years risk of T2DM through K-M method, as well.**

**Reply: Diagrams were added at the end of the figure.**

**Line 36: The healthy components of**

**Reply: A higher score is given to the consumption of healthy food groups.**

**Line 37: 1 for what?? I think it is 0.91**

**Reply: Number one is our reference.**

**Line 39: 1, 0.86◊ 0.86 0.93**

**Reply: The number 93 was changed to 0.93 and corrected.**

**Please recheck all the numbers you reported.**

**Reply: Agreed and corrected.**

**Keywords: please write them based on MeSH database.**

**Reply: Agreed and corrected.**

**Methods:
This section has been written well. However, please write this section according to the STROBE writing standard in terms of headings and subheadings.**

**Reply: Agreed and corrected.**

**Results:**

**Line 228-233: please recheck the numbers you reported in this paragraph.**

**Reply: Agreed and corrected.**

**The score of unhealthy in excessive amount food groups of the GDQS were conversely associated with T2D incidence in two crude and adjusted models [HR: 1, 0.89 (0.84-0.94). (page 14 line 237)**

**Reviewer #2:**

**Comments for the authors:**

**Thank you for your efforts. There are some major points that need improvement:
Abstract**

**1.The background of abstract provides no background, but just objective of the study.

Reply: Agreed and corrected.**

**Previous studies reported that focusing on healthy lifestyle especially high dietary quality is necessary for preventing type 2 diabetes (T2D).**

**Introduction**

**1. Line 48, the sentence “with a T2D incidence rate of 36.3% per 1000 person-years”: Incidence rates are typically expressed as the number of new cases per person-time (e.g., per 1000 person-years), not as a percentage. Percentages are usually used to express prevalence or risk, not incidence rates.**

**Reply: Following your comments this part has been revised.**

**In the Iranian population, there are >800,000 new cases of T2D per year, with a T2D incidence rate of 36.3 per 1000 person-years [3].( page 4 line 50-51)**

**2.Line 54, the sentence “Current meta-analysis studies suggest”: You are talking about studies while citing only one.**

**Reply: Agreed and corrected.**

**3.While the GDQS is introduced, a bit more detail on its composition and why it is considered a robust measure would be beneficial.**

**Reply: Food groups are divided into 16 healthy including kinds of fruits and vegetables, legumes, nuts and seeds, whole grain, fish, poultry, liquid oil, low fat dairy and egg; 7 unhealthy including processed meats, refined grains, sweets, sugar sweetened beverages, potato or cassava flour, juice, and deep-fried foods; and 2 unhealthy when consumed more than recommendation including red meat and high fat dairy. Page 4 line 61-65**

**4.The examples of Ref. 7&9 are not related that much to the topic, as of different study population. You must provide relevant example to highlight result of similar studies.**

**Reply: The GDQS index is a new measure with limited studies available. Since there are no research articles specifically examining this index in relation to diabetes, we relied on studies focused on related indices and diabetes occurrence.**

**Method**

**1.Not a common format to start the method section by ethics statement. Please provide details of study design.**

**Reply: Following your comment, this section has been revised.**

**The Tehran lipid and glucose study (TLGS) was performed prospectively on the 13th district of Tehran (the capital of Iran) residents to identify risk factors for non-communicable diseases [10, 11]. According to this study, the initial sample of 15005 participants (aged ≥3 years) were enrolled between 1999 and 2001. During the study, participants were followed every three years:** **wave 2 (2002-2005), wave 3 (2005-2008), wave 4 (2008-2011), wave 5 (2012-2015), wave 6 (2015-2018) to amend their demographic and health-related data, as well as identified diseases that have recently emerged. For this secondary analysis of subjects participating in waves 3 and 4 (baseline of our study), 8048 individuals aged ≥18 years were randomly selected to complete the dietary assessment based on age and sex distribution. Subjects with over- or under-reporting of energy intake (≥4,200 or <800 kcal/day) (n=780) were excluded [12], and a total of 7268 individuals with accessible biochemical, anthropometric, and dietary data were entered as the baseline population. They were tracked until phase 6; subjects who entered phase 3 or 4 in this study were respectively followed three and twice for the outcome measurement. Among these subjects, pregnant or lactating women, participants with T2D diagnosis based on fasting blood glucose (FBG) measurements or self-reported use of glucose-lowering medications at baseline (n=597), and subjects with missing data (n=208) were excluded. Also, subjects who did not provide follow-up data were excluded (n=515) from the study participants. As a result, 5948 subjects remained for analysis (Page 5-6 line 76-93).**

**2.Line 77, the sentence “this study was set up in district no.13 of Tehran to prevent non-communicable disease risk factors and their consequences”: I do not think that the study was directly designed to prevent NCDs.**

**Reply: Revisions were made to improve clarity in this section.**

**3.How did you choose to select 8048 participants? Please also explain more about randomization.**

**Reply: Revisions were made to improve clarity in this section.**

**The Tehran lipid and glucose study (TLGS) was performed prospectively on the 13th district of Tehran (the capital of Iran) residents to identify risk factors for non-communicable diseases [10, 11]. According to this study, the initial sample of 15005 participants (aged ≥3 years) were enrolled between 1999 and 2001. During the study, participants were followed every three years: wave 2 (2002-2005), wave 3 (2005-2008), wave 4 (2008-2011), wave 5 (2012-2015), wave 6 (2015-2018) to amend their demographic and health-related data, as well as identified diseases that have recently emerged. For this secondary analysis of subjects participating in waves 3 and 4 (baseline of our study), 8048 individuals aged ≥18 years were randomly selected to complete the dietary assessment based on age and sex distribution. Subjects with over- or under-reporting of energy intake (≥4,200 or <800 kcal/day) (n=780) were excluded [12], and a total of 7268 individuals with accessible biochemical, anthropometric, and dietary data were entered as the baseline population. They were tracked until phase 6; subjects who entered phase 3 or 4 in this study were respectively followed three and twice for the outcome measurement. Among these subjects, pregnant or lactating women, participants with T2D diagnosis based on fasting blood glucose (FBG) measurements or self-reported use of glucose-lowering medications at baseline (n=597), and subjects with missing data (n=208) were excluded. Also, subjects who did not provide follow-up data were excluded (n=515) from the study participants. As a result, 5948 subjects remained for analysis (Page 5-6 line 76-93).**

**4.You mentioned that total of 7268 individuals were selected as the baseline population, of whom 597 participants were excluded. Finally, you mentioned 5948 subjects remained for analysis, which is not logical. Also, please recheck all numbers mentioned in Figure 1. The summation of numbers included and excluded must be rechecked.**

**Reply: Revisions were made to improve clarity in this section.**

**5.I do not understand why the manuscript “Issues in analysis and presentation of dietary data” should be cited in the method section while explaining about inclusion of cases of your study (Ref. 10). I also doubt the necessity of citing your previous published work (Ref. 11&12) in method section.**

**Reply: Agreed and corrected**

**6.Line 100: Please define USDA.**

**Reply: The complete name added.**

**Due to the incompleteness and limited data on the nutrient content of cooked food items in the Iranian Food Composition Table (FCT), United States Department of Agriculture (USDA) data was used. The Iranian FCT was applied for national foods that could not be incorporated into the USDA portion size [2]. (Page 6 line 100-106)

Result
1.Better to include percentage of each frequency.**

**Reply: Modifications were implemented.**

**2.Consider providing more detailed subgroup analyses. For Table 1, post hoc analysis are recommended.**

**Reply: Following your comments post hoc analysis added (Page 12 line 105).**

**3. Include p-values for the differences in baseline characteristics to demonstrate statistical significance.**

**Reply: Modifications were implemented.**

**4. The approach to handling missing data is not discussed.**

**Reply: Individuals with missing data were excluded.**

**Discussion**

**Overall discussion section lacks logical flow and smooth transitions.**

**1.Line 256, the sentence “It seems that this association can help us to consume proper healthy food groups and limit unhealthy foods in excessive amounts”: Please provide more details, how it seems to help. Not a good beginning for discussion. Please elaborate more on the important findings and then turn to the application.**

**Reply: Revisions were made to improve clarity in this section.**

**We found an inverse association between the GDQS and the risk of T2D incidence among Tehranian adults. Our findings indicate that healthy eating can play a significant role in preventing and managing T2D. GDQS is a novel index that provides a more detailed breakdown of food group intake compared to similar indices. Limited studies available; therefore, we have used studies with similar topics here. Based on our knowledge, the association between GDQS and T2D has been studied only in the American population by Fang et al. in 2021. Our research was conducted in both male and female subjects in the Middle East, making this the first study to investigate this relationship in a developing country. Fang et al. indicated that a higher GDQS is associated with lower T2D risk, mostly due to a lower dietary intake of unhealthy foods in the US women [4]. In our analysis, high intakes of healthy food groups of the GDQS and low intakes of unhealthy in excessive amount food groups (high fat dairy and red meat) were strongly associated with a lower T2D risk; moreover, a higher score of the total unhealthy food groups of the GDQS or less consumption of unhealthy food groups, appeared to be negatively associated with T2D risk. It seems that the main negative role in the unhealthy subgroup is played by less consumption of red meat and high-fat dairy products. The research by He et al. indicates that there was an inverse association between a high GDQS and inadequacy of nutrients, as well as metabolic syndrome among the Chinese adults. They showed that individuals in the highest quintile of GDQS demonstrated a 21% lower probability of developing metabolic syndrome compared to those in the lowest GDQS group [8]. In a prospective study by Angulo et al, an increase in the GDQS, was associated with lower weight and WC gain in the Mexican women [9]. In Kang et al study GDQS was positively associated with maternal mid-upper arm circumference and BMI among pregnant women in rural Ethiopia, however, the result did not indicate any relationship with overweight or diet-related morbidity [7] page 14,15 line 261-285).**

**2.The third paragraph of discussion contains listing many references that have used GDQS index without providing any comparison with your results.**

**Reply: There are limited studies available; therefore, we have used studies with similar topics here.**

**3.Line 262: I do not understand why the sentence begins with “however”. What contrast dose it show?**

**Reply: Thanks for your comment. I discussed various healthy eating indexes (HEI, Mediterranean diet, etc.) with different score calculations. Ultimately, their patterns are similar, and we can compare them to some extent.**

**4.Line 274&276: The reference 8 should be mentioned as He et al. not Fang et al**

**Reply Modifications were implemented.**

**5.Line 273-5, the sentence “Based on our knowledge the relationship between GDQS and T2D has been studied only in the American population by Fang et al in 2021..”: This is incorrect. Other references:**
[**https://doi.org/10.1093/jn/nxab195**](https://doi.org/10.1093/jn/nxab195)
[**https://doi.org/10.1093/cdn/nzaa061_029**](https://doi.org/10.1093/cdn/nzaa061_029)
[**https://doi.org/10.1093/aje/kwy183**](https://doi.org/10.1093/aje/kwy183)

**Reply: The first and second suggested references are the same and are included in the study. The third reference pertains to diet quality indices that differ by GDQS. It was a helpful article, and based on your suggestion, we have included it in our article.**

**Also, Chen et al. demonstrated that adherence to a high-quality diet including the alternate Mediterranean diet (aMED), AHEI-2010, the Dietary Approaches to Stop Hypertension (DASH) diet, an overall plant-based diet index, and a healthful plant-based diet index was significantly associated with a lower risk of T2D in an Asian population [28] (page 19,20 line 296-299).**

**6.Lines 283-290: Lacks coherence and smooth transitions between different indexes.**

**Reply: The discussion section has been revised.**

**7.The discussion could benefit from a more detailed comparison of GDQS with other indices, specifically highlighting why GDQS might be more effective or accurate.**

**Reply: GDQS is a novel index that provides a more detailed breakdown of food group intake compared to other similar indices.**

**8.Lines 295-304: Seems more like dietary recommendation for diabetics, rather than discussion.**

**Reply: Thank you for your comment. there are some compelling studies that strongly support the recommendations for healthy and unhealthy food groups.**

**9.Provide clearer public health implications if the results**

**Reply: This section has been revised.**

**Examining this index in different regions of Iran with high prevalence of diabetes or other consequences will be beneficial to create a national index and guideline. In addition, with the help of this index, the nutritional status can be compared with other parts of the world. (Page 22 line 351-354)**

**10.In limitation, mention the generalizability and the need for further studies in diverse populations.**

**Reply: This section has been revised.**

**More studies are needed for generalizability in different populations. (page 22 Line 341-342)**

Thank you so much

Best regards

**Parvin Mirmiran, Prof.**
